# Tangeretin Inhibition of High-Glucose-Induced IL-1*β*, IL-6, TGF-*β*1, and VEGF Expression in Human RPE Cells

**DOI:** 10.1155/2020/9490642

**Published:** 2020-12-07

**Authors:** Dong Qin, Yan-rong Jiang

**Affiliations:** ^1^Henan Eye Institute, Henan Provincial Eye Hospital, People's Hospital of Zhengzhou University, Zhengzhou, China; ^2^Department of Ophthalmology, People's Hospital, Peking University, Beijing, China

## Abstract

Tangeretin, a natural compound extracted from citrus plants, has been reported to have antiproliferative, antidiabetic, anti-invasive, and antioxidant properties. However, the role of tangeretin in diabetic retinopathy (DR) is unknown. In the present study, we investigated whether tangeretin had any effect on the expression of interleukin 1 beta (IL-1*β*), interleukin 6 (IL-6), transforming growth factor beta 1 (TGF-*β*1), and vascular endothelial growth factor (VEGF) in human retinal pigment epithelial (RPE) cells under high-glucose (HG) conditions. Our results illustrated that HG levels induced IL-1*β*, IL-6, TGF-*β*1, and VEGF expression and that tangeretin significantly reduced HG-induced IL-1*β*, IL-6, TGF-*β*1, and VEGF expression in human RPE cells. Moreover, tangeretin efficiently inhibited the activation of the protein kinase B (Akt) signalling pathway in HG-stimulated RPE cells. Therefore, tangeretin may serve a role in the treatment of DR.

## 1. Introduction

Diabetic retinopathy (DR) is the leading cause of visual impairment and blindness among adults of working age [1]. Sustained hyperglycaemia plays an important role in the development of DR. The retinal pigment epithelial (RPE) cell is believed to contribute to the pathogenesis of DR. Proinflammatory cytokines, inflammatory mediators, and chemokines are also involved in the pathogenesis of DR. Previous studies have shown that high levels of interleukin 1 beta (IL-1*β*) are detected in the retinas of diabetic animals [2] and in the vitreous of patients with proliferative DR (PDR) [3]. In addition, high levels of interleukin 6 (IL-6) are also detected in the vitreous of patients with PDR or diabetic macular oedema [4–6].

Transforming growth factor beta (TGF-*β*) is reported to be involved in the differentiation, migration, proliferation, apoptosis, and accumulation of extracellular matrix molecules in various cell types [7]. TGF-*β*, a critical mediator and regulator, is associated with the pathophysiological processes of ocular tissue development or repair [8–11]. TGF-*β* is also believed to be involved in the development of DR. The TGF-*β* induction of vascular endothelial growth factor (VEGF) secretion by human RPE cells has a key role in neovascularisation in diabetic eye disease [12]. VEGF is a multifunctional molecule that is produced by some cell types in the retina in diabetes [13]. VEGF can trigger many retinal vascular changes caused by diabetes, including vascular leakage, capillary nonperfusion, and retinal neovascularisation [12, 13]. Hyperglycaemia in DR has been linked to the upregulation of VEGF.

Tangeretin, extracted from the peel of citrus fruits, has multiple pharmacological properties, including antioxidant, antiasthmatic, anti-inflammatory, and neuroprotective properties [14–16]. It was reported that tangeretin had potent neuroprotective effects against pilocarpine-induced seizures [17] and attenuated brain injury in a rat model [18]. However, the effect of tangeretin on DR has not been investigated. Therefore, the present study shows that high-glucose (HG) levels induce the expression of IL-1*β*, IL-6, TGF-*β*1, and VEGF in human RPE cells. HG also activates the phosphorylation of protein kinase B (Akt) in human RPE cells, and tangeretin can inhibit the phosphorylation of Akt under HG conditions. In addition, tangeretin significantly inhibits the HG-induced expression of IL-1*β*, IL-6, TGF-*β*1, and VEGF in human RPE cells. Thus, tangeretin may serve a role in the treatment of DR.

## 2. Materials and Methods

### 2.1. Reagents

Anti-Akt was obtained from Cell Signaling Technology (Danvers, MA, USA). IL-1*β*, IL-6, TGF-*β*1, and VEGF enzyme-linked immunosorbent assay (ELISA) kits were purchased from Abcam (Cambridge, MA, USA). LY294002 was obtained from Sigma-Aldrich/Merck KGaA.

### 2.2. Cell Culture

Human RPE cell line (ARPE-19; CRL-2302) was obtained from the American Type Culture Collection (Manassas, VA, USA). The cells were cultured in Dulbecco's Modified Eagle Medium (DMEM, Gibco, Grand Island, NY, USA); the medium was supplemented with 10% foetal bovine serum, 100 ng/ml streptomycin, and 100 U/ml penicillin. The cells were maintained at 37°C in a humidified incubator of 5% CO_2_.

### 2.3. MTT Assay

The MTT assay was performed for cell viability. The human RPE cells were plated into 96-well plates at a density of 1 × 10^4^/well. After treatment with different concentrations of tangeretin (0, 5, 10, 20, and 30 *μ*M) for 24 h, the MTT reagents were added to each well and incubated for 4 h. Then, the medium was removed, and dimethyl sulfoxide (DMSO) was added to dissolve the formazan crystals. The absorbance was read at 490 nm using a microplate reader.

### 2.4. Real-Time Polymerase Chain Reaction (PCR) Analysis

Total RNAs were extracted from human RPE cells using a TRIzol reagent kit. The cDNA was prepared using a RevertAid First Strand cDNA Synthesis Kit (Fermentas, St. Leon-Roth, Germany). Real-time PCR was performed in triplicates on a Real-Time System (Bio-Rad, Munich, Germany). Each reaction contained 2.5 *μ*l cDNA, 12.5 *μ*l Maxima SYBR Green qPCR Master Mix (Fermentas, Waltham, MA, USA), and specific primers (0.3 *μ*M each), with a final volume of 25 *μ*l. The primers were as follows: human IL-1*β*, forward 5′-GGA CAA GCT GAG GAA GAT GC-3 and reverse 5′-TCC ATA TCC TGT CCC TGG AG-3′; human IL-6, forward 5′-TGG CTG AAA AAG ATG GAT GCT-3′ and reverse 5′-TCT GCA CAG CTC TGG CTT GT-3′; human TGF-*β*1, forward 5′-GCC AGG ATA TGA GTT TGG GA-3′ and reverse 5′-GGG TGC ATG TCT GCT CCT GT-3′; and human VEGF, forward 5′-AAG GAG GAG GGC AGA ATC AT-3′ and reverse 5′-ATC TGC ATG GTG ATG TTG GA-3′. The reaction conditions were 95°C for 30 s, followed by 39 cycles of 95°C for 5 s and 60°C for 30 s. The RNA expression was normalized to the level of GAPDH mRNA.

### 2.5. Western Blot Analysis

After treatment, human RPE cells were lysed in radioimmunoprecipitation assay (RIPA) buffer supplemented with phenylmethylsulphonyl fluoride (PMSF) protease inhibitors. The protein concentration was quantified using a bicinchoninic acid assay (BCA). The protein samples were loaded on 10% SDS-PAGE gels and transferred to polyvinylidene fluoride (PVDF) membranes (Millipore, Billerica, MA, USA). They were processed for analysis using an enhanced chemiluminescence (ECL) detection system (Amersham, Arlington Heights, IL, USA). The dilutions for the primary antibodies were as follows: the anti-p-Akt was diluted at 1 : 2000, and the antitotal Akt was diluted at 1 : 1000.

### 2.6. ELISA Analysis

After treatment, the samples were collected. The protein levels of IL-1*β*, IL-6, TGF-*β*1, and VEGF in the culture supernatants were determined using IL-1*β*, IL-6, TGF-*β*1, and VEGF ELISA kits according to the manufacturer's instructions.

### 2.7. Statistical Analysis

Statistical analysis was performed using a one-way analysis of variance (ANOVA) followed by Tukey's test. All data are expressed as mean ± standard deviation (SD). They were analysed using SPSS 17.0 (SPSS, Chicago, IL, USA). A *p* value < 0.05 was considered statistically significant.

## 3. Results

### 3.1. Effect of Tangeretin on RPE Cell Viability

**Figure 1:**
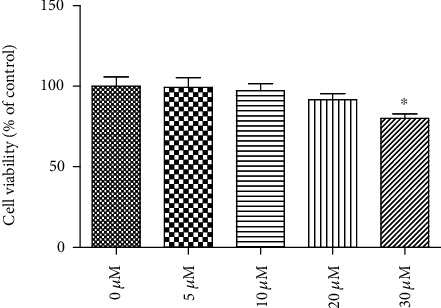
Effect of tangeretin on human RPE cell viability. The cells were incubated with different concentrations of tangeretin (0, 5, 10, 20, and 30 *μ*M) for 24 h. The MTT assay was then performed to measure cell viability of human RPE cells. ^∗^*p* < 0.05 vs. control group (0 *μ*M).

To evaluate the cytotoxicity effect of tangeretin on RPE cells, the cells were incubated with different concentrations of tangeretin (0, 5, 10, 20, and 30 *μ*M) for 24 h. The MTT assay showed that tangeretin (30 *μ*M) caused the decrease in cell viability; however, tangeretin at a concentration of 5, 10, and 20 *μ*M did not affect the viability of RPE cells (Figure 1).

### 3.2. Induction of IL-1*β*, IL-6, TGF-*β*1, and VEGF in HG Conditions in RPE Cells

**Figure 2:**
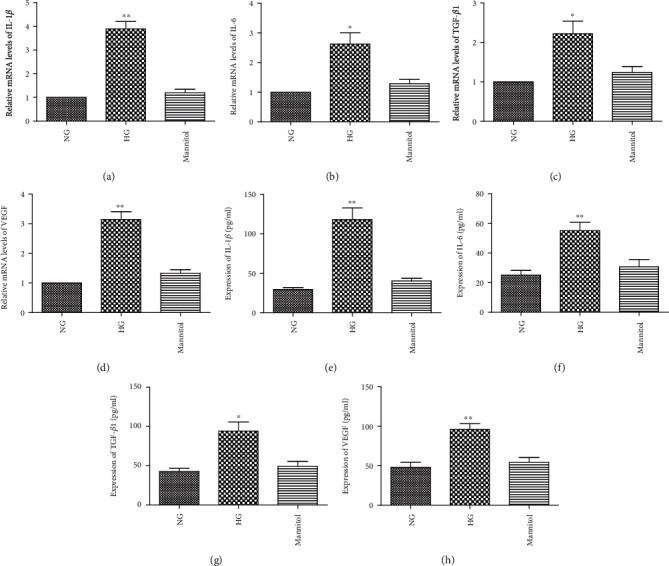
HG levels induced the expression of IL-1*β*, IL-6, TGF-*β*1, and VEGF in RPE cells. RPE cells were exposed to NG (5.5 mM), HG (30 mM), and mannitol (24.4 mM) for 24 h before the expressions of IL-1*β*, IL-6, TGF-*β*1, and VEGF were measured. When compared with NG, a real-time PCR and an ELISA kit analysis showed that the mRNA (a–d) and protein (e–h) levels of IL-1*β*, IL-6, TGF-*β*1, and VEGF were upregulated in response to HG. The data shown represents the mean ± SD of three independent experiments. ^∗^*p* < 0.05 versus NG; ^∗∗^*p* < 0.01 versus NG.

We examined the expression of IL-1*β*, IL-6, TGF-*β*1, and VEGF in HG conditions. The human RPE cells were cultured in DMEM containing normal glucose (NG; 5.5 mM) and high glucose (30 mM) and were exposed for 24 h. Real-time PCR and ELISA kit data revealed an increased mRNA level in IL-1*β*, IL-6, TGF-*β*1, and VEGF in the cells under the HG condition (Figures 1(a)–1(d)). An increased protein level in IL-1*β*, IL-6, TGF-*β*1, and VEGF was also observed in the cells under the HG condition (Figures 2(e)–2(h)).

### 3.3. Effect of HG and Tangeretin on Akt Signalling Pathways in Human RPE Cells

**Figure 3:**
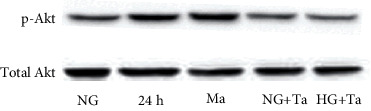
The effect of HG and tangeretin activated the phosphorylation of Akt in RPE cells. RPE cells were stimulated with HG (30 mM) or 24.4 mmol/l mannitol for 24 h. Cell lysates were immunoblotted with anti-p-Akt and anti-Akt antibodies. RPE cells were pretreated with 20 *μ*M tangeretin for 1 h and then incubated with HG for 24 h for the assay of Akt phosphorylation. Ta: tangeretin; Ma: mannitol.

To examine the effect of HG and tangeretin on Akt signalling pathways, the human RPE cells were cultured in DMEM containing either NG (5.5 mM) or HG (30 mM) and were exposed for 10 min or 20 min, with or without pretreatment with 20 *μ*M tangeretin for 30 min. A western blot analysis showed that HG can activate the phosphorylation of Akt in RPE cells. The phosphorylation of Akt was blocked by pretreatment with tangeretin under HG conditions for 20 min (Figure 3).

### 3.4. Tangeretin and Akt Inhibitor LY294002 Suppress the HG-Induced Expression of IL-1*β*, IL-6, TGF-*β*1, and VEGF in Human RPE Cells

**Figure 4:**
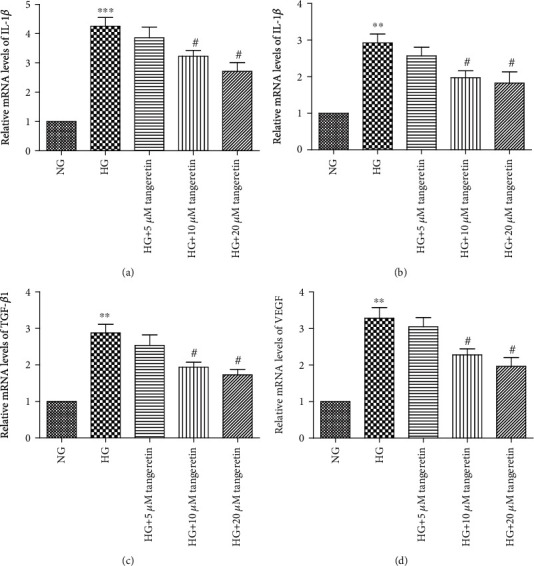
Tangeretin inhibited the mRNA expression of IL-1*β*, IL-6, TGF-*β*1, and VEGF in RPE cells. RPE cells were pretreated with various concentrations of tangeretin (5 *μ*M, 10 *μ*M, and 20 *μ*M) for 1 h and then stimulated by the addition of HG (30 mM) for 24 h. A real-time PCR analysis was performed to assess the expression of IL-1*β*, IL-6, TGF-*β*1, and VEGF. Tangeretin significantly decreased the HG-induced mRNA (a–d) level of IL-1*β*, IL-6, TGF-*β*1, and VEGF in RPE cells in a dose-dependent manner. Values are expressed as the mean ± SD of three independent experiments. ^∗∗^*p* < 0.01 versus NG; ^∗∗∗^*p* < 0.001 versus NG; ^#^*p* < 0.05 versus HG.

**Figure 5:**
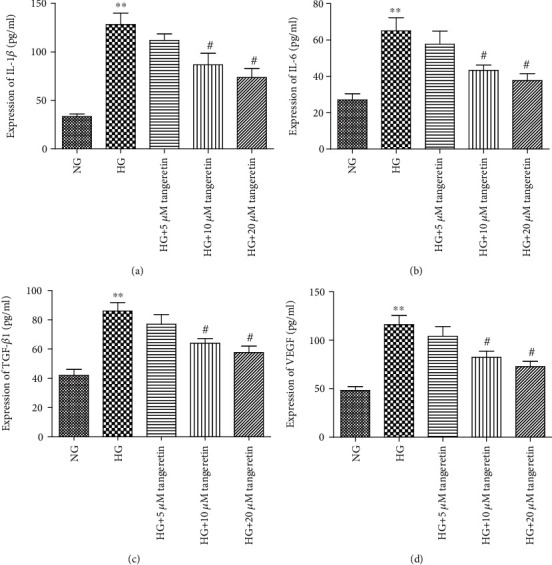
Tangeretin decreased the protein level of IL-1*β*, IL-6, TGF-*β*1, and VEGF in RPE cells. After pretreatment with various concentrations of tangeretin (5 *μ*M, 10 *μ*M, and 20 *μ*M) for 1 h, RPE cells were stimulated by the addition of HG (30 mM) for 24 h. An ELISA kit analysis was performed to assess the protein level of IL-1*β*, IL-6, TGF-*β*1, and VEGF. Tangeretin significantly decreased the HG-induced protein (a–d) levels of IL-1*β*, IL-6, TGF-*β*1, and VEGF in RPE cells in a dose-dependent manner. Values are expressed as the mean ± SD of three independent experiments. ^∗∗^*p* < 0.01 versus NG; ^#^*p* < 0.05 versus HG.

**Figure 6:**
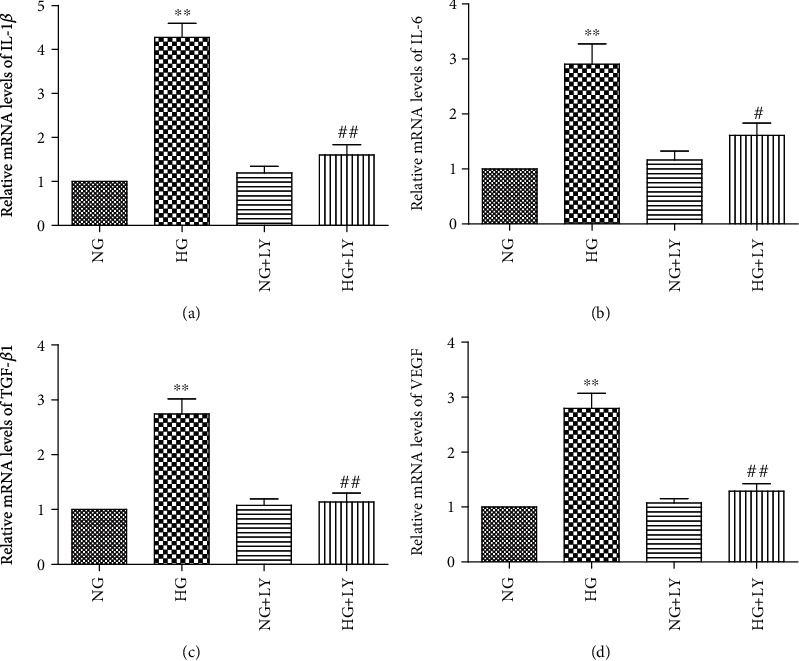
Inhibition of Akt signalling downregulated HG-induced IL-1*β*, IL-6, TGF-*β*1, and VEGF mRNA expression in RPE cells. RPE cells were preincubated for 1 h with 10 *μ*M of LY294002 and then stimulated by HG (30 mM) for 24 h. The IL-1*β*, IL-6, TGF-*β*1, and VEGF mRNA levels were determined using real-time PCR. LY294002 significantly decreased the HG-induced mRNA (a–d) levels of IL-1*β*, IL-6, TGF-*β*1, and VEGF in RPE cells. The data shown represent the mean ± SD of three independent experiments. ^∗∗^*p* < 0.01 versus NG; ^#^*p* < 0.05 versus HG; ^##^*p* < 0.01 versus HG.

**Figure 7:**
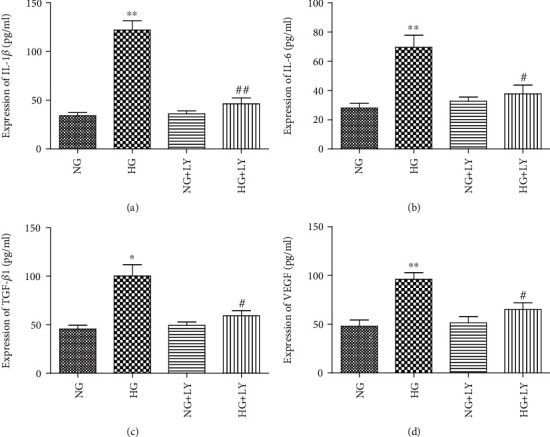
The PI3K/Akt signalling pathway mediated the protein level of IL-1*β*, IL-6, TGF-*β*1, and VEGF in RPE cells under high-glucose conditions. RPE cells were preincubated for 1 h with 10 *μ*M of LY294002 and then stimulated by HG (30 mM) for 24 h. The IL-1*β*, IL-6, TGF-*β*1, and VEGF protein levels were determined using the ELISA kit. LY294002 significantly decreased the HG-induced protein (a–d) levels of IL-1*β*, IL-6, TGF-*β*1, and VEGF in RPE cells. The data shown represent the mean ± SD of three independent experiments. ^∗^*p* < 0.05 versus NG; ^∗∗^*p* < 0.01 versus NG; ^#^*p* < 0.05 versus HG; ^##^*p* < 0.01 versus HG. LY: LY294002.

Having found that HG promoted the expression of IL-1*β*, IL-6, TGF-*β*1, and VEGF and induced the phosphorylation of Akt in human RPE cells, we then examined whether tangeretin had any effect on the HG-induced expression of IL-1*β*, IL-6, TGF-*β*1, and VEGF. In addition, we examined whether the activation of the phosphorylation of Akt plays a vital role in the HG-induced expression of IL-1*β*, IL-6, TGF-*β*1, and VEGF in RPE cells. Using an ELISA kit and a real-time PCR assay, the HG-induced expression of IL-1*β*, IL-6, TGF-*β*1, and VEGF was shown to be inhibited by tangeretin in a dose-dependent manner in human RPE cells (Figures 4 and 5). Meanwhile, the pretreatment of RPE cells with LY294002 inhibited the HG-induced expression of IL-1*β*, IL-6, TGF-*β*1, and VEGF (Figures 6 and 7).

## 4. Discussion

Hyperglycaemia is one of the most important initiators of the pathogenesis of DR. Sustained hyperglycaemia can upregulate growth factors, cytokines, and other molecules. Studies have demonstrated that the levels of IL-1*β* and IL-6 are upregulated in the vitreous of patients with PDR [2–6]. In this study, we chose IL-1*β* and IL-6 as our target genes, examining whether tangeretin had any effect on the HG-induced expression of IL-1*β* and IL-6. We demonstrated that HG significantly increased the induction of IL-1*β* and IL-6 in RPE cells when they were exposed to 30 mM glucose for 24 h. Interestingly, tangeretin significantly decreased the expression of IL-1*β* and IL-6 in human RPE cells under the condition of 30 mM glucose in a dose-dependent manner, which suggested that tangeretin could suppress cytokine secretion under the HG condition in human RPE cells.

TGF-*β*, a multifunctional cytokine, regulates critical cell biological actions, such as migration, differentiation, and apoptosis. TGF-*β* is reported to be one of the most important ligands in the pathological processes of fibrotic diseases in the retina, including PDR, proliferative vitreoretinopathy (PVR), and retinopathy of prematurity (ROP) [19, 20]. In addition, TGF-*β* is believed to contribute to the contraction of subretinal and epiretinal membranes in patients with PVR and PDR [20]. VEGF is a potent angiogenic stimulator of neovascularisation, and it promotes vascular permeability. VEGF plays a vital role in the pathogenesis of DR [21–23]. High levels of VEGF in both human and animal samples are reported to be associated with the development and progression of DR [24–26]. Our data illustrated that the expression of TGF-*β*1 and VEGF was upregulated in RPE cells when exposed to 30 mM glucose for 24 h. In addition, tangeretin significantly reduced the HG-induced expression of TGF-*β*1 and VEGF in human RPE cells in a dose-dependent manner. The findings indicated that tangeretin could suppress the expression of TGF-*β*1 and VEGF in the human RPE cell under the HG condition.

The phosphoinositide 3-kinase (PI3K)/Akt signalling pathway plays an important role in DR and in numerous cellular functions, including proliferation, migration, invasion, adhesion, metabolism, and survival [27]. The PI3K pathway is associated with the formation of normal blood vessels [28]. Studies have reported that the inhibition of the Akt pathway could inhibit pathological vascularisation [29] and many tumour types [30]. Our previous study showed that HG activated the phosphorylation of Akt, and inhibition of the PI3K/Akt signalling pathway could inhibit the expression of extracellular matrix molecules under HG conditions in RPE cells [31]. In this study, we found that inhibition of Akt abolished HG-induced IL-1*β*, IL-6, TGF-*β*1, and VEGF expression in human RPE cells. The findings of the present study also showed that 30 mM glucose also activated the phosphorylation of Akt, and 20 *μ*M tangeretin significantly inhibited the phosphorylation of Akt in RPE cells under the HG condition. These findings indicated that tangeretin may inhibit the expression of IL-1*β*, IL-6, TGF-*β*1, and VEGF through the Akt signalling pathway.

In future research, the role of tangeretin should be investigated in animal models in vivo. In addition, oxidative stress and other pathways involved in inflammation and activated by high glucose such as JNK, P38 MAPK, and NF-*κ*B should be conducted. These are the limitations of the present study. In summary, it has been reported that tangeretin has multifunctional properties, including anti-invasive, antiproliferative, antimetastatic, antidiabetic, and antioxidative properties. However, the role of tangeretin in DR is unclear. The present study demonstrated that HG levels induced IL-1*β*, IL-6, TGF-*β*1, and VEGF expression and the phosphorylation of Akt and that tangeretin significantly reduced the HG-induced expression of IL-1*β*, IL-6, TGF-*β*1, and VEGF in human RPE cells. Thus, tangeretin significantly reduced cytokine secretion in HG environments and extends our knowledge in the treatment of diabetic retinopathy.

## Data Availability

The data used to support the findings of this study are available from the corresponding author upon request.

## Additional Points

*Highlights*. High glucose induced the expression of IL-1*β*, IL-6, TGF-*β*1, and VEGF in human RPE cells. Tangeretin inhibited the activation of the protein kinase B (Akt) signalling pathway in high-glucose-stimulated human RPE cells. Tangeretin significantly inhibited high-glucose-induced IL-1*β*, IL-6, TGF-*β*1, and VEGF expression in human RPE cells.
